# Target expression is a relevant factor in synthetic lethal screens

**DOI:** 10.1038/s42003-022-03746-6

**Published:** 2022-08-19

**Authors:** Iman J. Schultz, Herjan J. T. Coelingh Bennink

**Affiliations:** Pantarhei Oncology B.V., Boulevard 17, 3707BK Zeist, The Netherlands

**Keywords:** CRISPR-Cas9 genome editing, Target identification

**arising from** Y. Gilad et al. *Commun. Biol.* 10.1038/s42003-021-01929-1 (2021).

Synthetic lethal screens support the discovery of novel cancer drug targets^1^. In a recent issue of *Communications Biology*, Gilad et al.^2^ performed a synthetic lethal CRISPR Cas9 dropout screen in the human, estrogen receptor positive breast cancer (BC) cell line MCF-7. They aimed to identify targets that increased the sensitivity of the MCF-7 cells to the small molecule inhibitor SI-12, which targets SRC-3, an essential transcriptional cofactor of the estrogen receptor. A key finding of their screen indicated that targeting certain olfactory receptors (ORs) might confer anti-tumor effects in BC. However, these ORs, and a number of other hits, are not expressed in MCF-7 cells, calling into question the setup of the screen and warranting the inclusion of transcriptome data into the analysis pipeline of genetic screens.

The identification of novel drug targets is an important pillar in expanding the treatment options for patients with cancer. Through their screen, Gilad et al. identified the olfactory receptors *OR4D6* and *OR5I1*, next to a number of other genes, as potential targets in patients with breast cancer^2^. Interestingly, other studies have implicated olfactory receptors (ORs) in cancer biology in general^3^, and also specifically in BC^4,5^. The studies by Weber et al.^4^ and Masjedi et al.^5^ performed transcriptome analyses of both BC cell lines and BC tumor tissues to assess OR expression, and many ORs were detected. Surprisingly though, both studies did not detect *OR4D6* or *OR5I1*, the ORs identified in the screen by Gilad et al., in any of the BC related biological samples, which included MCF-7. We further looked into the expression of these two ORs in cancer cell lines by interrogating online expression databases (The Human Protein Atlas (https://www.proteinatlas.org/), the Broad Institute Cancer Dependency Map (https://depmap.org/portal/) (version 21Q1)), which confirmed absence of expression of these ORs in MCF-7 cells. Further investigation indicated that a number of other targets identified in the CRISPR Cas9 screen by Gilad et al. were also not expressed in this cell line (*NDNF*, *S1PR1*) according to the online databases.

To explore whether the synthetic lethal CRISPR Cas9 results extended beyond the MCF-7 cell line, Gilad et al. exposed additional BC cell lines (T-47D, BT-474, ZR-75-1 and MDA-MB-231) to SI-12 after transient silencing of a number of the identified targets, including *OR4D6*, using siRNAs. Although less potent than in MCF-7 cells, silencing *OR4D6* still sensitized three of these cell lines to SI-12 treatment, leading the authors to conclude that this gene may be an interesting therapeutic target in BC in general. Still, the data by Weber et al. and Masjedi et al. showed absence of expression of this OR in these additional BC cell lines used by Gilad et al., which was confirmed by the data in the online expression repositories.

A possible explanation for the therapeutic effects of silencing the identified targets that apparently lack expression in the investigated cancer cell lines, could be that their expression is induced upon exposure to SI-12. However, Gilad et al. exposed cells to SI-12 after CRISPR Cas9 or siRNA mediated gene interference. Therefore, it appears the CRISPR Cas9 screen selected a number of targets with no biological role in MCF7 cells. This suggests that, despite the ample attention Gilad et al. paid to the setup and execution of their screen, the internal experimental controls may have been insufficient to accurately identify valid targets. In this light, it is surprising that the siRNA validation experiments Gilad et al. performed also indicated that silencing the ‘non-expressed’ targets conferred anti-tumor effects. It has been described that both CRISPR Cas9 and RNAi technologies, although to different extents, suffer from off-target effects^6,7^.

When high-throughput functional genetic screens found their way into biomedical research, a lot of effort was put into optimizing the technical aspects and data analysis. However, a prerequisite for assessing a gene’s biological role in a particular context is expression at physiologically relevant levels, an aspect that is equally important assigning value to when performing functional screens. Therefore, it is highly recommended that under the experimental conditions of a genetic screen, the full transcriptome of the biological test sample is determined. This should be integrated in the data analysis pipeline to exclude transcripts with irrelevant expression levels. Also, excluding non-expressed targets prevents wasting time on the validation of such ‘hits’. In this regard, publicly available transcriptome data from resources like The Human Protein Atlas and the DepMap consortium offer a good starting point, although these pertain to untreated samples.

The study by Gilad et al., in our opinion, illustrates the need to integrate target gene expression data into the analysis pipeline of a functional genetic screen. In addition, it showcases the susceptibility of siRNA-based target validation to off-target effects, since silencing the non-expressed targets resulted in a phenotypic effect. An important recommendation for siRNA-based validation is performing rescue experiments^6^, which were not performed by Gilad et al. Also, methodologies have been developed to distinguish between on- and off-target effects in siRNA- and CRISPR Cas9-based screens^8^. Stringent follow-up of all recommendations that have been put forward over the years to increase the validity of the output of functional genetic screens should result in a more robust identification of promising drug targets. Therefore, while the results of other studies have attested to an interesting role for ectopically expressed olfactory receptors in breast cancer, to our opinion the data presented by Gilad et al. in this respect need to be revisited.

## Data Availability

The data that support the findings of this study are available at https://www.proteinatlas.org/ and https://depmap.org/portal/.
